# Supplementary material for: Impact of Changes in Time Left Alone on Separation-Related Behaviour in UK Pet Dogs
Source: Animals (Basel). 2022 Feb 15;12(4):482. doi: 10.3390/ani12040482 (PMC8868415; doi:10.3390/ani12040482)
Supplement: Supplementary file 1 [file animals-12-00482-s001.zip › animals-1523603-supplementary.pdf]

animals

Supplementary material

Impact of Changes in Time Left Alone on Separation-Related Behaviour in UK Pet Dogs

Naomi D. Harvey *, Robert M. Christley, Kassandra Giragosian, Rebecca Mead, Jane K. Murray, Lauren Samet, Melissa M. Upjohn and Rachel A. Casey

Canine Behaviour and Research, Dogs Trust, EC1V 7RQ London, UK; robert.christley@dogstrust.org.uk (R.M.C.); kassandra.giragosian@dogstrust.org.uk (K.G.); rebecca.mead@dogstrust.org.uk (R.M.); jane.murray@dogstrust.org.uk (J.K.M.); lauren.samet@dogstrust.org.uk (L.S.); melissa.upjohn@dogstrust.org.uk (M.M.U.); rachel.casey@dogstrust.org.uk (R.A.C.)

* Correspondence: naomi.harvey@dogstrust.org.uk

Citation: Harvey, N.D.; Christley, R.M.; Giragosian, K.; Mead, R.; Murray, J.K.; Samet, L.; Upjohn, M. M.; Casey, R.A. Impact of Changes in Time Left Alone on Separation-Related Behaviour in UK Pet Dogs. *Animals* **2022**, *12*, 482. https://doi.org/10.3390/ani12040482

Academic Editor(s): Paola Maria Valsecchi and Lynette A. Hart

Received: 8 December 2021
Accepted: 10 February 2022
Published: 15 February 2022

Publisher's Note: MDPI stays neutral with regard to jurisdictional claims in published maps and institutional affiliations.

Copyright: © 2022 by the authors. Licensee MDPI, Basel, Switzerland. This article is an open access article distributed under the terms and conditions of the Creative Commons Attribution (CC BY) license (https://creativecommons.org/licenses/by/4.0/).

This paper focusses on data from two questions that asked about time the dog was left alone without human company (number of days in the past 7 days/an average week in February that the dog was left for at least 5 minutes; longest period of time left alone in the past 7 days/an average week in February) and two questions on whether owners had observed any of a pre-defined list of separation-related behaviours (SRBs) when their dog was left, and when about to be left, (in the past 7 days/an average week in February).

The questions are shown below, as they appeared in the two surveys. Within the surveys, piping was used to insert the dogs' names into the questions. Here, the name "Ted" is used to example this.

Survey One

This survey required owners to describe the dog's behaviour/management during the last 7 days (i.e., during the first phase of lockdown), and also based upon recall during early/mid-February 2020 to provide a pre-pandemic baseline before people had started to alter their lifestyle to avoid virus transmission. The questions analysed in this paper were:

1. On how many days per week was Ted left home alone for at least five minutes (without human company)?

	Not at all	1 day	2 days	3 days	4 days	5 days	6 days	7 days	Don't know/can't remember
During an average week in early/mid-February 2020	□	□	□	□	□	□	□	□	□
During the last 7 days	□	□	□	□	□	□	□	□	□

2. What was the longest period that Ted was left alone without human company?

	Not at all	Less than 5 minutes	5-19 minutes	20-59 minutes	1 or more hours but less than 3 hours	3 or more hours but less than 6 hours	6 or more hours	Don't know/can't remember	NA - Ted was not left on his/her own
During an average week in early/mid-February 2020	□	□	□	□	□	□	□	□	□
During the last 7 days	□	□	□	□	□	□	□	□	□

3. Have any of the following applied to Ted, when he/she was <u>about to be left</u> without human company? He/she…

Please select all that apply.

	Early/mid-February 2020	The last 7 days
Vocalised (barked / howled / pined / whined / cried)	□	□
Paced around, or turned in circles or chased his/her tail	□	□
Chewed or destroyed non-food items other than toys	□	□

Scratched / damaged around the door, skirting boards, windows or entrance to the house	◻	◻
Scratched / damaged furniture	◻	◻
Urinated and/or defecated inside the home	◻	◻
None of the above	◻	◻
NA - Ted was not left on his/her own	◻	◻

Other behaviours (please specify):

4. Have any of the following applied to Ted, when he/she was left without human company? He/she…

Please select all that apply.

	Early/mid-February 2020	The last 7 days
Vocalised (barked / howled / pined / whined / cried)	◻	◻
Paced around, or turned in circles or chased his/her tail	◻	◻
Chewed or destroyed non-food items other than toys	◻	◻
Scratched / damaged around the door, skirting boards, windows or entrance to the house	◻	◻
Scratched / damaged furniture	◻	◻
Urinated and/or defecated inside the home	◻	◻
None of the above	◻	◻
NA - Ted was not left on his/her own	◻	◻

Other behaviours (please specify):

Survey Two

In the follow-up October 2020 survey, the owners to describe their dog's behaviour/management within the last 7 days. The questions appeared as follows:

1. On how many days per week was Ted left home alone for at least five minutes (without human company)?

	Not at all	1 day	2 days	3 days	4 days	5 days	6 days	7 days	Don't know/can't remember
During the last 7 days	▫	▫	▫	▫	▫	▫	▫	▫	▫

2. What was the longest period that Ted was left alone without human company?

	Not at all	Less than 5 minutes	5-19 minutes	20-59 minutes	1 or more hours but less than 3 hours	3 or more hours but less than 6 hours	6 or more hours	Don't know/can't remember	NA - Ted was not left on his/her own
During the last 7 days	▫	▫	▫	▫	▫	▫	▫	▫	▫

3. Have any of the following applied to Ted, when he/she was <u>about to be left</u> without human company? He/she…

Please select all that apply.

	The last 7 days
Vocalised (barked / howled / pined / whined / cried)	▫
Paced around, or turned in circles or chased his/her tail	▫
Chewed or destroyed non-food items other than toys	▫
Scratched / damaged around the door, skirting boards, windows or entrance to the house	▫
Scratched / damaged furniture	▫
Urinated and/or defecated inside the home	▫
None of the above	▫
NA - Ted was not left on his/her own	▫
Other behaviours (please specify):	

4. Compared with how Ted behaved in this situation in early/mid-February, his/her behaviour when <u>about to be left</u> alone over the last 7 days has been…
   - o The same as before
   - o Different to before
   - o N/A – Ted was not left on his/her own at both timepoints
   - o N/A – I did not own Ted in February 2020

   If applicable, please describe how this has changed:

5. Have any of the following applied to Ted, when he/she was <u>left</u> without human company? He/she…

Please select all that apply.

	The last 7 days
Vocalised (barked / howled / pined / whined / cried)	◻
Paced around, or turned in circles or chased his/her tail	◻
Chewed or destroyed non-food items other than toys	◻
Scratched / damaged around the door, skirting boards, windows or entrance to the house	◻
Scratched / damaged furniture	◻
Urinated and/or defecated inside the home	◻
None of the above	◻
NA - Ted was not left on his/her own	◻
Other behaviours (please specify):	

6. Compared with how Ted behaved in this situation in early/mid-February, his/her behaviour when he/she was left alone over the last 7 days has been…
   - The same as before
   - Different to before
   - N/A – Ted was not left on his/her own at both timepoints
   - N/A – I did not own Ted in February 2020

   If applicable, please describe how this has changed:

Restrictions in place across the study period

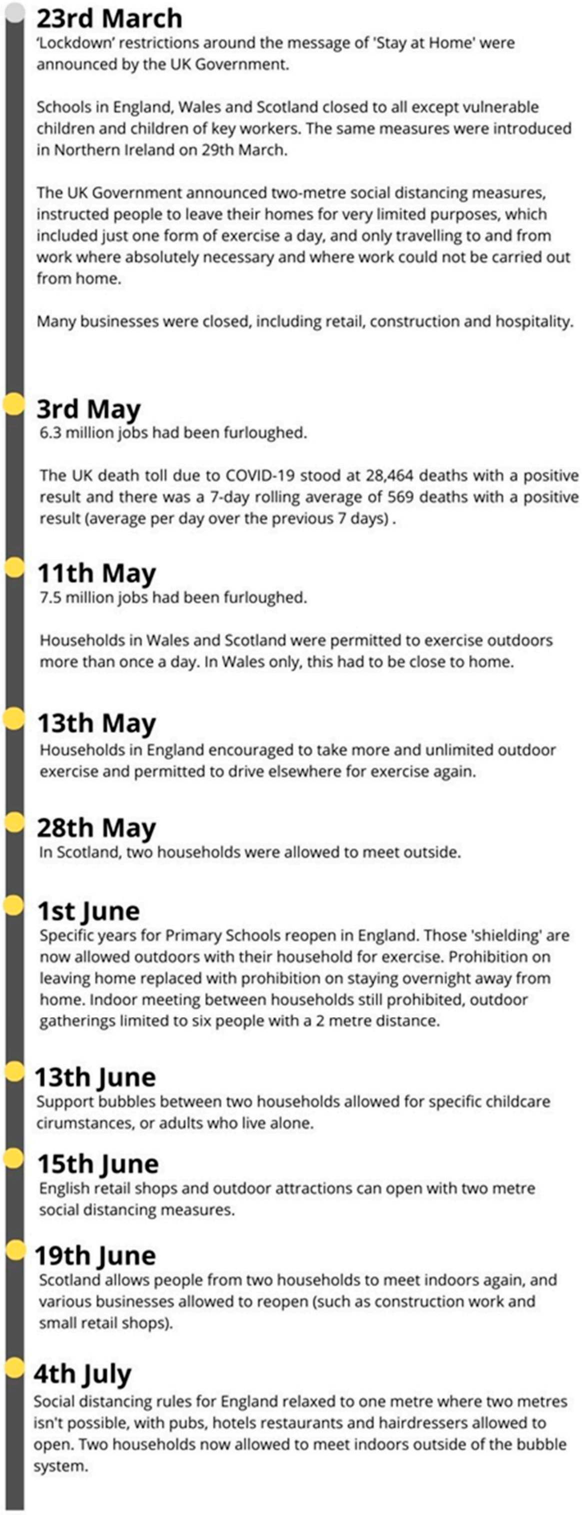

The first survey contained responses from dog owners during the period of 4th May to 3rd July, 2020. At the start of this period (between 4th and 12th of May), the UK was under its strictest 'lockdown' restrictions which began on 23rd March, with people only allowed to leave homes for limited reasons, including shopping for food, exercise once per day, medical need and travelling for work when absolutely necessary (Figure S1).

The follow-up survey was open for completion between the 10th October and 2nd November 2020, coinciding with a local 'tier-system' for COVID-19 restrictions which was introduced on 12th October [23]. The tier-system meant that different areas were under differing levels of restrictions, either 'medium', 'high' or 'very high'. At this time, workplaces that had previously been closed (including hospitality, retail, building work and beauty salons) were now open again, and people were once again allowed to meet others indoors in most places unless their local Tier specifically prohibited it.

Figure S1. Timeline providing an example of the COVID-19 restrictions in place across the UK during the May/June survey period (shown with yellow points). Dates for different steps taken by devolved nations (Wales, Scotland, Northern Ireland and England) differed but steps were largely taken at similar times, so key examples are provided only. Although the dates in this figure end at July 4th, no major changes occurred at a national level between then and the time of the second survey in October.
